# Frequency and Clinicohistopathological Concordance of Psoriasis and Psoriasiform Dermatitis: A Tertiary Care Study

**DOI:** 10.7759/cureus.115980

**Published:** 2026-09-09

**Authors:** Ram Raghuvanshi, Shashikant, Mustafa Ali, Hukam Singh Meena, Chakshu Sukheja, Iti Gupta

**Affiliations:** 1 Pathology, Virendra Kumar Sakhlecha Government Medical College, Neemuch, IND; 2 Pathology, Dr. Laxminarayan Pandey Government Medical College, Ratlam, IND; 3 Pathology, Atal Bihari Vajpayee Government Medical College, Vidisha, IND; 4 Pathology, Mahatma Gandhi Memorial Medical College and Maharaja Yeshwantrao Hospital, Indore, IND

**Keywords:** concordance, erythematous plaque, histopathology, psoriasiform dermatitis, psoriasis, scaly papules, skin biopsy

## Abstract

Objectives

The present study was conducted to determine the frequency of psoriasis and psoriasiform dermatitis among patients attending the Dermatology Outpatient Department (OPD) of a tertiary care hospital. The study also aimed to assess the concordance between the clinical and histopathological diagnoses of these lesions, and to characterize the key epidermal and dermal histopathological features of confirmed cases of psoriasis.

Materials and methods

This study was conducted over a period of one year and included all patients attending the Dermatology OPD with a clinical diagnosis of psoriasis or psoriasiform dermatitis who were subsequently advised to undergo a skin biopsy for histopathological examination in the Department of Pathology. Cases were enrolled after applying the predefined inclusion and exclusion criteria.

Results

A total of 45 patients were enrolled in the study. Histopathological analysis revealed that psoriasis was the most common diagnosis, accounting for 77.8% (n=35) of cases. Dermatitis was identified in 6.7% (n=3) and hypertrophic lichen planus in 6.6% (n=3) of cases. Psoriasis also demonstrated the highest clinicohistopathological concordance, with 86.8% (n=33) of clinically diagnosed cases being confirmed on histopathological examination.

Conclusion

This study highlights the diverse histopathological spectrum and frequency of psoriasis and psoriasiform lesions. Furthermore, it concludes that the clinical diagnosis shows good concordance with the histopathological diagnosis in the majority of cases.

## Introduction

Psoriasis and psoriasiform dermatitis constitute the largest group of papulosquamous skin disorders, characterized by erythematous, scaly papules and plaques [1,2].

Psoriasis is a chronic inflammatory skin disease in which genetic predisposition and autoimmune mechanisms play pivotal roles in its pathogenesis [3]. It is usually divided into pustular and non-pustular types clinically. Overall, psoriasis vulgaris is the most common type of psoriasis, accounting for approximately 90% of all cases. It is characterized by well-demarcated, erythematous plaques covered with silvery-white scales, which commonly involve the trunk, limbs, and scalp. Other uncommon types include Guttate psoriasis, erythrodermic psoriasis, palmoplantar psoriasis, generalized/localised pustular psoriasis, and impetigo herpetiformis [4-6].

Psoriasiform dermatitis encompasses a group of inflammatory skin disorders that exhibit clinical and histopathological features resembling those of psoriasis. Some of the important entities include seborrheic dermatitis, chronic eczematous dermatitis, atopic dermatitis, allergic contact dermatitis, irritant contact dermatitis, nummular dermatitis, lichen simplex chronicus, hypertrophic lichen planus, and tinea pedis [7].

The clinical and histological features of psoriasis and other psoriasiform dermatitis often overlap, resembling various dermatological conditions and exhibiting diverse clinical presentations, thereby making the actual diagnosis challenging [8]. Therefore, the present study aimed to determine the frequency of psoriasis and psoriasiform dermatitis among patients with clinically suspected psoriasis or psoriasiform dermatitis who underwent skin biopsy at a tertiary care center. The secondary objective was to assess the clinicohistopathological concordance between the clinical and histopathological diagnoses and characterize the key histopathological features of confirmed psoriasis.

## Materials and methods

This was a prospective descriptive study conducted in the departments of Pathology and Dermatology of the Mahatma Gandhi Memorial (MGM) Medical College and Maharaja Yeshwantrao (M.Y.) Hospital, in Indore, Madhya Pradesh, from December 1, 2023, to November 30, 2024. The study was approved by the Ethics and Scientific Review Committee, MGM Medical College and M.Y. Hospital (approval number: EC/MGM/Nov-2023/221).

Eligibility criteria

Patients aged >18 years who attended the Dermatology outpatient department (OPD) with a clinical diagnosis of psoriasis or psoriasiform dermatitis and subsequently underwent skin biopsy for histopathological examination in the Department of Pathology were included in the study after obtaining written informed consent. Patients who did not provide informed consent, those with poorly preserved tissue specimens, and cases with inadequate tissue samples for histopathological evaluation were excluded from the study.

Sample size calculation

The sample size was calculated a priori using Hulley’s formula for a correlation coefficient: $N = \left[\frac{Z_{\alpha} + Z_{1-\beta}}{C}\right]^2 + 3$, where N is the required sample size, Zα is the standard normal deviate corresponding to a two-sided significance level of 5% (1.96), Z1−β is the standard normal deviate corresponding to 80% statistical power (0.84), and C = 0.5 × $\ln\left(\frac{1+r}{1-r}\right)$, where r is the anticipated correlation coefficient. An anticipated r value of 0.41 was selected as a medium effect-size estimate in the absence of prior population-specific data. This yielded a minimum required sample size of approximately 45 participants. As the primary variables were categorical clinical and histopathological diagnoses, this sample-size approach was not optimally aligned with the subsequent agreement analysis and is acknowledged as a methodological limitation.

Data collection

After obtaining written informed consent, each patient was clinically examined by a dermatologist. Relevant clinical details, including the site, size, colour, morphology, and distribution of the lesion(s), and demographic details were recorded. Following clinical evaluation and documentation of the relevant clinical findings in the Department of Dermatology, a lesional punch or superficial and deep shave biopsy was performed in patients with a clinical diagnosis of psoriasis or psoriasiform dermatitis. The biopsy specimens were subsequently submitted to the Department of Pathology for histopathological examination.

Gross and microscopic examination

A proper gross description including tissue size, presence or absence of epidermis, color, presence or absence of hair, and alterations to the epidermal surface was noted. Samples were then processed, sectioned, and stained with hematoxylin and eosin. Slides were independently examined by two pathologists with 5-10 years of experience, both blinded to the clinical diagnosis at the time of histopathological evaluation. Psoriasis was diagnosed based on characteristic features including parakeratosis, elongation of rete ridges, suprapapillary thinning, hyperkeratosis, acanthosis, Munro microabscesses, and dilated/proliferated papillary dermal blood vessels with perivascular lymphocytic infiltration. Differential diagnoses, including dermatitis, hypertrophic lichen planus, pityriasis lichenoides chronica, parapsoriasis, and pityriasis rosea, were diagnosed according to established histopathological criteria. Difficult or discordant cases between the two pathologists were resolved through discussion with a senior professor in the department.

Data analysis

The collected data were entered into Microsoft Excel (Microsoft Corporation, Redmond, Washington, United States) and analysed using IBM SPSS Statistics for Windows, version 23.0 (IBM Corp., Armonk, New York, United States). Continuous variables were expressed as mean ± standard deviation (SD), while categorical variables were presented as frequencies and percentages. The association between clinical and histopathological diagnoses was assessed using the Fisher-Freeman-Halton exact test. Cohen's kappa was calculated to assess agreement between clinical and histopathological diagnoses beyond chance. A p-value of <0.05 was considered statistically significant.

## Results

A total of 45 patients were enrolled over the one-year study period after fulfilling the inclusion and exclusion criteria. The majority of study participants (n= 16; 35.6%) belonged to the 31-40 years age group, followed by those in the 18-30 years age group (n=15; 33.3%). Male patients constituted the majority of the study population (n=32; 71%), while female patients accounted for 29% of the sample (n=13).

Among the study participants, the most commonly reported clinical history was of red, raised, or itchy skin lesions, either generalized or localized, observed in 33.3% (n=15) of individuals. This was followed by scaly or white scaly patches/lesions and non-specific or generalized skin lesions, each reported by 13.3% (n=6) of participants (Table 1).

**Table 1:** Distribution of clinical characteristics among study participants (N=45)

Clinical history	Frequency	Percentage
Red/raised/itchy skin lesions (generalized or localized)	15	33.30%
Scaly/white scaly patches or lesions	6	13.30%
Erythema/erythematous plaques	4	8.90%
Hyperpigmented lesions	2	4.40%
Fissuring/cracking of hands or feet	3	6.70%
Nail changes with hyperpigmentation	1	2.20%
Exfoliation/peeling of skin with or without itching	3	6.70%
Multiple plaques or patches (circular/red, etc.)	3	6.70%
Non-specific/generalized skin lesions	6	13.30%

The majority of study participants (n= 38; 84.4%) were clinically diagnosed with psoriasis, making it the most prevalent clinical diagnosis in the study population. Other diagnoses were considerably less common, with dermatitis accounting for 6.6% (n=3) of cases. Hypertrophic lichen planus, pityriasis lichenoides chronica, small-plaque parapsoriasis, and tinea pedis each accounted for 2.2% (n=1) of cases (Table 2).

**Table 2:** Distribution of clinical diagnoses among the study participants (N=45)

Clinical diagnosis	Frequency	Percentage
Hypertrophic Lichen Planus	1	2.2
Dermatitis	3	6.6
Pityriasis Lichenoid Chronica	1	2.2
Psoriasis	38	84.4
Small plaque parapsoriasis	1	2.2
Tinea Pedis	1	2.2

Histopathological analysis revealed that psoriasis was the most common diagnosis, observed in 77.8% (n=35) of cases. Dermatitis and hypertrophic lichen planus were identified in 6.7% (n=3) and 6.6% (n=3) of cases, respectively. Less frequently observed histopathological diagnoses included pityriasis lichenoides chronica (n=2, 4.4%), parapsoriasis (n=1, 2.2%), and pityriasis rosea (n=1, 2.2%) (Table 3).

**Table 3:** Distribution of histopathological diagnoses among the study participants (N=45)

Histopathological diagnosis	Frequency	Percentage
Dermatitis	3	6.7
Hypertrophic Lichen Planus	3	6.6
Parapsoriasis	1	2.2
Pityriasis Lichenoid Chronica	2	4.4
Pityriasis rosea	1	2.2
Psoriasis	35	77.8

A statistically significant association was observed between the clinical and histopathological diagnoses using the Fisher-Freeman-Halton exact test (p = 0.002). Cohen's kappa demonstrated moderate agreement between clinical and histopathological diagnoses beyond chance (κ = 0.47, 95% CI: 0.14-0.80). Overall clinicohistopathological concordance was observed in 37 of 45 cases (82.2%). Among the 38 clinically diagnosed cases of psoriasis, 33 (86.8%) were confirmed histopathologically (Table 4).

**Table 4:** Agreement between clinical and histological diagnoses Fisher-Freeman-Halton exact test: p = 0.002; Cohen's kappa: κ = 0.47 (95% CI: 0.14–0.80)

Clinical diagnosis		Histopathological diagnosis						Total
		Dermatitis	Hypertrophic Lichen Planus	Parapsoriasis	Pityriasis Lichenoid Chronica	Pityriasis rosea	Psoriasis	
Dermatitis	n	2	0	0	0	0	1	3
	%	66.7%	0.0%	0.0%	0.0%	0.0%	33.3%	100.0%
Hypertrophic Lichen Planus	n	0	1	0	0	0	0	1
	%	0.0%	100.0%	0.0%	0.0%	0.0%	0.0%	100.0%
Pityriasis Lichenoid Chronica	n	0	0	0	1	0	0	1
	%	0.0%	0.0%	0.0%	100.0%	0.0%	0.0%	100.0%
Psoriasis	n	1	2	1	0	1	33	38
	%	2.6%	5.3%	2.6%	0.0%	2.6%	86.8%	100.0%
Small plaque parapsoriasis	n	0	0	0	1	0	0	1
	%	0.0%	0.0%	0.0%	100.0%	0.0%	0.0%	100.0%
Tinea Pedis	n	0	0	0	0	0	1	1
	%	0.0%	0.0%	0.0%	0.0%	0.0%	100.0%	100.0%
Total	n	3	3	1	2	1	35	45
	%	6.7%	6.7%	2.2%	4.4%	2.2%	77.8%	100.0%

Among the 35 histopathologically confirmed cases of psoriasis, parakeratosis was the most frequently observed feature, present in 32 cases (91.4%). This was followed by elongated rete ridges in 31 cases (88.6%) and suprapapillary thinning in 28 cases (80.0%). Hyperkeratosis and acanthosis were each observed in 25 cases (71.4%). Munro microabscesses were present in 22 cases (62.9%), while spongiosis and hypogranulosis were each observed in 21 cases (60.0%) (Table 5, Figure 1).

**Table 5:** Epidermal and dermal histopathological features in psoriasis (N=35)

Histopathological features in Psoriasis	Frequency	Percentage
Hyperkeratosis	25	72%
Parakeratosis	32	92%
Munroe Microabscess	22	64%
Suprapapillary Thinning	28	80%
Elongated Rete Ridges	31	88%
Acanthosis	25	72%
Blood vessels proliferation	25	72%
Lymphocytic aggregation	31	88%
Spongiosis	21	60%
Hypogranulosis	21	60%

**Figure 1:**
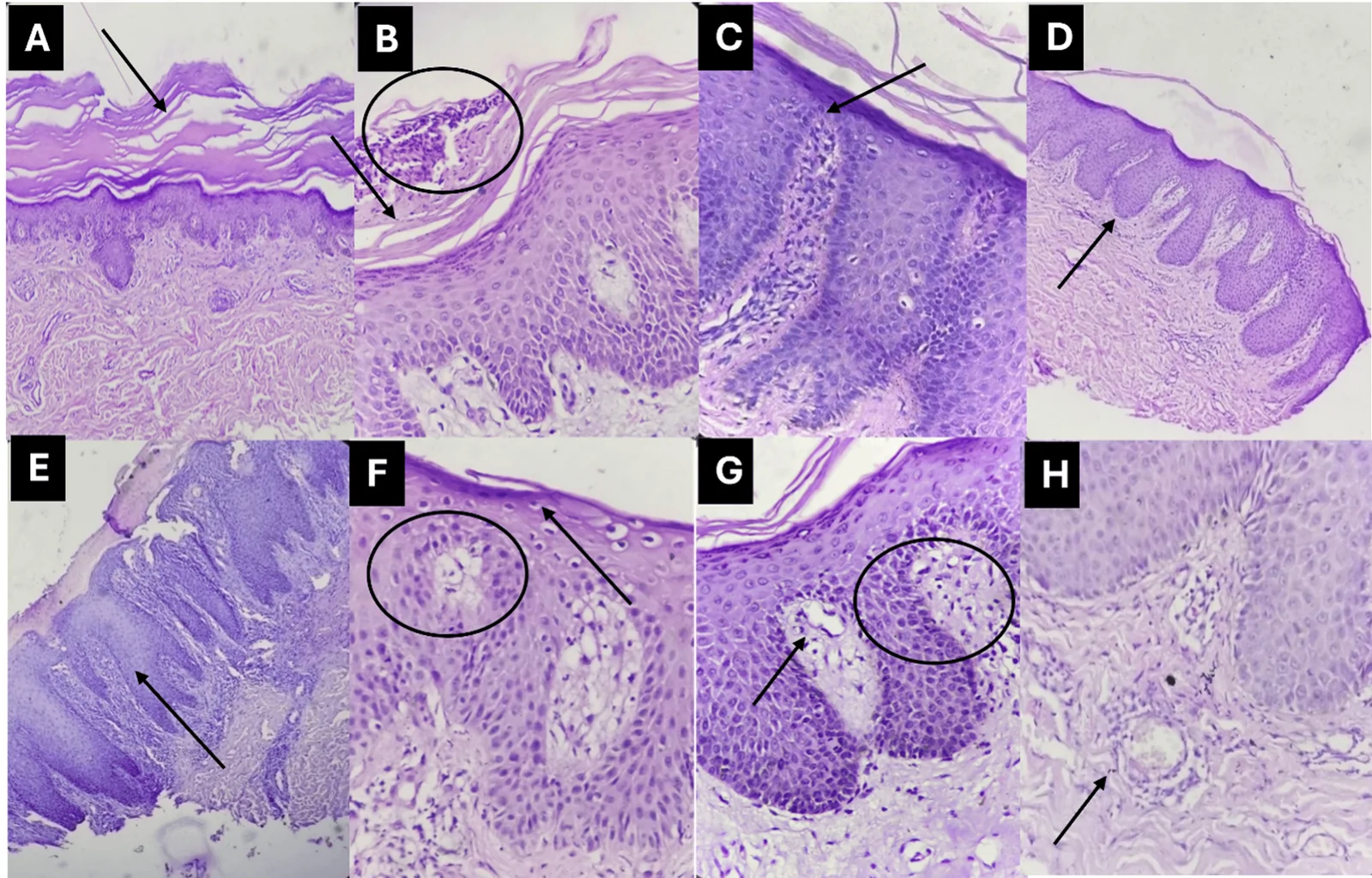
Epidermal and dermal histologic features of psoriasis (A) Hyperkeratosis: thickened stratum corneum with orthokeratotic keratin (arrow) (H&E stain, 100× magnification); (B) Parakeratosis: retention of nuclei in the stratum corneum (arrow), Munro's microabscess: presence of neutrophilic collection in stratum corneum (Circle) (H&E stain, 100× magnification); (C) Suprapapillary thinning (arrow) (H&E, 400× magnification); (D) Elongation of rete ridges (arrow) (H&E stain, 100× magnification); (E) Acanthosis: Increased thickness of the stratum spinosum (arrow) (H&E stain, 100× magnification); (F) Spongiosis: abnormal accumulation of fluid in the epidermis (circle), hypogranulosis: decreased thickness  of granular layer (arrow) (H&E stain, 400× magnification); (G) Papillary dermis showing proliferated blood vessels (arrow) and lymphocytic aggregation (circle) (H&E stain, 400× magnification); (H) Perivascular lymphocytic infiltration (arrow) (H&E stain, 400× magnification) H&E: hematoxylin and eosin

## Discussion

Accurate diagnosis of psoriasis and other psoriasiform dermatitis is essential for appropriate treatment, prognostic assessment, and optimal patient management. As many of these papulosquamous disorders exhibit overlapping clinical features, histopathological examination remains the gold standard for establishing a definitive diagnosis and differentiating among these conditions.

In the present study, the majority of the study participants belonged to the age group of 31-40 years. Similar findings were reported by Reddy et al. [9]. In contrast, Veldurthy et al. [10] and Younas and Haque [11] observed that the age group of 20-30 years was the most commonly affected. In the present study, the 18-30 years age group was the second most common; however, the frequency was almost comparable to that of the 31-40 years age group. Male patients were more frequently affected than female patients in the present study. Similar findings were reported by Reddy and Krishna [9] and D' Costa and Bharambe [12], who also observed a male predominance.

In the present study, psoriasis was by far the most common diagnosis on both clinical and histopathological examination. Similar findings were reported by Nagpal et al. [13] and Venna et al. [14]. However, Ward et al. reported that psoriasis and psoriasiform dermatitis occurred with nearly equal frequency [15]. This difference from the present study may be attributed to geographical and ethnic variations in the study population. In the study by D' Costa and Bharambe, 97.52% of the clinical diagnoses were concordant with the histopathological diagnoses [12]. Similarly, Younas and Haque reported a clinicohistopathological concordance of 76.30% [11]. This was comparable with the findings of the present study, where the clinicohistopathological concordance was 82.2%. Notably, two patients clinically diagnosed as psoriasis were reclassified as hypertrophic lichen planus on histopathological examination, reflecting the well-recognized clinical and histopathological overlap between these two papulosquamous conditions [16].

Parakeratosis was the most common epidermal histopathological finding, observed in 92% of patients, while lymphocytic aggregation was the most common dermal histopathological finding, present in 88.6% of psoriasis cases. The differences in the most common epidermal and dermal histopathological features reported across various studies may be attributed to variations in the stage of the lesion at the time of biopsy, biopsy site, histologic subtype of psoriasis, and geographical and ethnic differences among the study populations [9,17,18].

Limitations

Despite these findings, the study has several limitations. The relatively small sample size of 45 patients and single-center, biopsy-selected design may limit statistical precision and generalizability, and referral/selection bias cannot be excluded. The marked diagnostic imbalance, with very small numbers in several non-psoriasis categories, limits reliable disease-specific concordance estimates. Although histopathological assessment was independently performed by two blinded pathologists, formal interobserver agreement was not quantified. The sample-size calculation based on Hulley’s correlation formula was not optimally aligned with the categorical agreement analysis and represents a methodological limitation. Lesion stage, biopsy site, and prior treatment were not systematically controlled. Long-term follow-up was not performed, and immunohistochemical investigations such as Ki-67 were not performed. These factors should be considered when interpreting the findings and their applicability beyond this tertiary-care population.

## Conclusions

Psoriasis was the most common histopathological diagnosis among patients clinically suspected to have psoriasis or psoriasiform dermatitis, with parakeratosis and lymphocytic aggregation being the most frequently observed epidermal and dermal histopathological features, respectively. A high clinicohistopathological concordance was observed in the study, indicating that clinical diagnosis is reliable in most cases. However, histopathological examination remains essential for confirming the diagnosis and differentiating psoriasis from other psoriasiform dermatoses with overlapping clinical features. Therefore, clinicopathological concordance should be considered the cornerstone for the accurate diagnosis and appropriate management of psoriasis and psoriasiform dermatitis.
